# Supplementary material for: Costs of Palliative Care in Oncological and Non-Oncological Patients with Different Types of Ambulatory-Based Attention: Cost-Study Protocol
Source: Diseases. 2024 Oct 5;12(10):243. doi: 10.3390/diseases12100243 (PMC11507158; doi:10.3390/diseases12100243)
Supplement: Supplementary file 1 [file diseases-12-00243-s001.zip › diseases-3176995-Supplementary Files S1 and S2.pdf]

S1_Cuestionario_Paciente_Spanish

Por favor completa TODOS LOS DATOS que se solicitan a continuación.

¡Gracias! :)

A continuación encontrará un cuestionario relacionado con los costes del cuidado paliativo que está recibiendo en este momento.
Le solicitamos lea atentamente cada pregunta y responda con total sinceridad, no hay. respuestas correctas.
Sus respuestas nos ayudarán a conocer más sobre los costes de sus cuidados, para poder aportar datos a las fuentes científicas que estudian el tema a nivel mundial.

Digitador

- ◯ AHPB
- ◯ CD
- ◯ MTB
- ◯ PACIENTE
- ◯ CUIDADOR

(Iniciales del digitador de los datos)

Fase

- ◯ Pilotaje - PRUEBA
- ◯ Reclutamiento - RECLUTAMIENTO

(Fase de recolección de datos)

Centro

- ◯ Clinic
- ◯ Mollet
- ◯ Granollers
- ◯ Vendrell
- ◯ Otro

(Se trata del Centro de referencia del Paciente.)

Detalle Otro Centro

____
(Si es Otro , por favor detallar)

ID Paciente

____
(Anote número de H. Clínica del Paciente)

Nombre Paciente

____

Entrevista a paciente hecha CON CUIDADOR

- ◯ Yes
- ◯ No

Razón de Entrevista CON CUIDADOR

- ◯ Paciente frágil (i.e., enfermo, edad)
- ◯ Paciente con demencia
- ◯ Paciente solicita que sea entrevistado el cuidador
- ◯ Paciente fallecido
- ◯ Otros

Si razón de razón de ENTREVISTA CON CUIDADOR es OTRO, detallar:

____
(Anotación breve)

S1 Datos Socio-Demográficos

S11 Fecha Entrevista Paciente

____

S12 Género Paciente

◯ Femenino
◯ Masculino
◯ Otros

S13 Si la respuesta es otros, especificar

____

S14 Fecha nacimiento Paciente

____

Edad Paciente

____

S141 ¿Paciente ha fallecido?

◯ No data
◯ Yes
◯ No

S142 Si el/la paciente ha fallecido, anote la fecha

____

S15 Código Postal Residencia Paciente

____

S16 País Origen Paciente

◯ España
◯ Extranjero

S17 Si es Extranjero	◯ Europa norte<br>◯ Europa centro<br>◯ Europa este<br>◯ Europa sur<br>◯ Resto Europa<br>◯ Reino Unido / Irlanda<br>◯ África<br>◯ Asia / Australia / Oceanía<br>◯ América Norte<br>◯ América Centro<br>◯ América Sur
S18 Estado Civil Paciente	◯ Soltero/a<br>◯ Casado/a<br>◯ Divorciado/a - Separado/a<br>◯ Viudo/a<br>◯ Desconocido
S19 Número de personas que habitan en su vivienda	__________________________________
S110 Nivel de estudios Paciente	◯ No sabe leer o escribir<br>◯ Educación Primaria incompleta<br>◯ Educación Primaria completa (EGB)<br>◯ Primera etapa de enseñanza secundaria, con o sin título<br>◯ Estudios de Bachillerato<br>◯ Enseñanzas profesionales de grado medio o equivalente (FP)<br>◯ Enseñanzas profesionales de grado superior o equivalente<br>◯ Estudios universitarios o equivalente<br>◯ Desconocido
S111 Situación laboral actual del Paciente	◯ Trabaja por cuenta ajena<br>◯ Trabaja por cuenta propia<br>◯ Jubilado o pensionista (anteriormente ha trabajado)<br>◯ Parado/a y ha trabajado antes<br>◯ Parado/a y busca su primer empleo<br>◯ Estudiante<br>◯ Incapacitado/a para trabajar<br>◯ Trabajo doméstico no remunerado<br>◯ Otra situación<br>◯ Desconocido

S112 Si trabaja por cuenta ajena, seleccione la categoría laboral en la cual se encuentra el Paciente	◯ Directores y gerentes<br>◯ Técnicos y profesionales científicos e intelectuales de la salud y la enseñanza<br>◯ Otros técnicos y profesionales científicos e intelectuales<br>◯ Técnicos; profesionales de apoyo<br>◯ Empleados de oficina que no atienden al público<br>◯ Empleados de oficina que atienden al público<br>◯ Trabajadores de los servicios de restauración y comercio<br>◯ Trabajadores de los servicios de salud y el cuidado de las personas<br>◯ Trabajadores de los servicios de protección y seguridad<br>◯ Trabajadores cualificados en el sector agrícola, ganadero, forestal y pesquero<br>◯ Trabajadores cualificados de la construcción, excepto operadores de máquinas<br>◯ Trabajadores cualificados de las industrias manufactureras, excepto operadores de instalaciones y máquinas<br>◯ Operadores de instalaciones y máquinas fijas, y monitores<br>◯ Conductores y operadores de maquinaria móvil<br>◯ Trabajadores no cualificados en servicios (excepto transportes)<br>◯ Peones de la agricultura, pesca, construcción, industrias manufactureras y transportes<br>◯ Ocupaciones militares<br>◯ Desconocido
S113 Especifique la profesión del Paciente si no puede clasificarla en la entrevista, en las categorías anteriores.	__________________________________<br>(PARA EL ENTREVISTADOR)
S114 Disponibilidad de un plan privado de salud	◯ Sí<br>◯ No<br>◯ NS/NC
S115 ¿Cuál es el importe mensual neto de los ingresos totales del hogar en la actualidad?	◯ Menos de 500 €<br>◯ De 500 € a menos de 1.000 €<br>◯ De 1.000 € a menos de 1.500 €<br>◯ De 1.500 € a menos de 2.000 €<br>◯ De 2.000 € a menos de 2.500 €<br>◯ De 2.500 € a menos de 3.000 €<br>◯ De 3.000 € a menos de 5.000 €<br>◯ De 5.000 € a menos de 7.000 €<br>◯ De 7.000 € a menos de 9.000 €<br>◯ 9.000 € y más €<br>((ingresos percibidos por todos los miembros del hogar, aporten o no estos ingresos o parte de los mismos para sufragar los gastos del hogar))

S116 Porcentaje aportación prescripción farmacéutica (medicamentos prescritos y recogidos en farmacia)	◯ TSI 001 - 0% (exento)<br>◯ TSI 002 - 10% (límite máximo 8,23 €/mes)<br>◯ TSI 002 - 10% (límite máximo 18,52 €/mes)<br>◯ TSI 003 - 40%<br>◯ TSI 004 - 50%<br>◯ TSI 005 - 60%<br>◯ TSI 005 - 60% (límite máximo 61,75 €/mes)<br>◯ TSI 006 - 30%<br>◯ NS/NC

S2 Sobre los Cuidados Paliativos

S217 ¿Cuál es el diagnostico principal por el que inició el programa de cuidados paliativos?	◯ Diagnóstico oncológico<br>◯ Diagnostico respiratorio<br>◯ Diagnostico cardiovascular<br>◯ Otros
S218 Especifique el Diagnóstico	__________________________________
S219 ¿Desde qué fecha está en el programa de cuidados paliativos?	__________________________________
S220 ¿En qué contexto recibe los cuidados paliativos?	◯ Visitas externas hospitalarias y o ambulatorios centro de atención primaria+ ingresos hospitalarios puntuales<br>◯ Hospitalización intercalado con cuidados paliativos domiciliarios<br>◯ Cuidados paliativos domiciliarios + ingresos hospitalarios puntuales<br>◯ Combinación de diferentes contextos de cuidados paliativos

S321 Detalle de visitas, equipos y procedimientos

S3211 ¿Ha hecho visitas al médico del CAP?	◯ Yes<br>◯ No
S32111 ¿Cuántas visitas (aproximadamente) ha realizado al médico del CAP desde el ingreso en CP?	__________________________________
S3212 ¿Ha hecho visitas al médico Especialista de CP?	◯ Yes<br>◯ No
S32121 ¿Cuántas visitas (aproximadamente) ha realizado al médico Especialista en CP desde el ingreso en CP?	__________________________________
S3213 ¿Ha hecho visitas a la Enfermera de CP?	◯ Yes<br>◯ No
S32131 ¿Cuántas visitas (aproximadamente) ha realizado a la Enfermera de CP desde el ingreso en CP?	__________________________________
S3214 ¿Ha hecho visitas al Terapista Ocupacional / Físico?	◯ Yes<br>◯ No

S32141 ¿Cuántas visitas (aproximadamente) ha realizado a Terapia Ocupacional/ Física desde el ingreso en CP?	__________________
S3215 ¿Ha hecho visitas al Hospital de Día?	◯ Yes<br>◯ No
S32151 ¿Cuántas visitas (aproximadamente) ha realizado a Hospital de Día desde el ingreso en CP?	__________________
S3216 ¿Ha recibido visitas de PADES?	◯ Yes<br>◯ No
S32161 ¿Cuántas visitas (aproximadamente) le ha realizado PADES desde el ingreso en CP?	__________________
S3217 ¿Ha sido ingresado/hospitalizado desde que está en el programa de CP?	◯ Yes<br>◯ No
S32171 ¿Cuántas veces (aproximadamente) ha requerido ser ingresado/hospitalizado desde el ingreso en CP?	__________________
S3221 ¿Le han hecho procedimientos ambulatorios desde que está en el programa de CP?	◯ Yes<br>◯ No
S32211 Procedimientos ambulatorios, ¿cuántos (aproximadamente) le han hecho desde el ingreso a CP?	__________________<br>(Procedimientos ambulatorios (p.e., infusiones de medicamentos, procedimientos manejo dolor))
S322111 Detalle los Procedimientos Ambulatorios	__________________<br>(De la lista anotada)
S3222 Equipos médicos, ¿ha requerido desde que está en el programa de CP?	◯ Yes<br>◯ No
S322211 Detalle los Equipos Médicos	__________________<br>(De la lista anotada)
S32221 Equipos médicos ¿cuántos ha requerido el paciente desde el ingreso a CP?	__________________<br>(Equipos médicos (ayudas para movilidad, ayudas para caminar, férulas, nebulizadores))
S3223 Tests diagnósticos o de imagen, ¿ha requerido desde que está en el programa de CP?	◯ Yes<br>◯ No
S32231 Test Diagnósticos o de Imagen ¿cuántos ha requerido el paciente desde el ingreso a CP?	__________________<br>(Test Dx / Imagen (extracciones sanguíneas, examen de orina, medicina nuclear, radiología))

S322311 Detalle los Test Diagnósticos / Imágenes

____
(De la lista anotada)

S323 Medicación de CP con receta en el último mes

S3231 ¿Está tomando analgésicos/anti-inflamatorios?
◯ Yes
◯ No
((Paracetamol, ibuprofeno, otros))

S32311 Detalle los anti-inflamatorios con posología

____
(De la lista anotada)

S3232 ¿Está tomando analgésicos opioides?
◯ Yes
◯ No
((Morfina, fentanilo, buprenorfina, tapentadol, codeína))

S32321 Detalle los analgésicos opioides con posología

____
(De la lista anotada)

S3233 ¿Toma otros analgésicos coadyuvantes?
◯ Yes
◯ No
((Pregabalina, gabapentina, duloxetina))

S32331 Detalle los analgésicos coadyuvantes con posología

____
(De la lista anotada)

S3234 ¿Toma protectores de estómago?
◯ Yes
◯ No
((Omeprazol, pantoprazol))

S32341 Detalle los protectores de estómago con posología

____
(De la lista anotada)

S3235 ¿Toma procinéticos?
◯ Yes
◯ No
((Metoclopramida, levogastrol))

S32351 Detalle los procinéticos con posología

____
(De la lista anotada)

S3236 ¿Toma laxantes?
◯ Yes
◯ No
((Movicol, L. magnesia, dulcolaxo, enemas))

S32361 Detalle los laxantes con posología

____
(De la lista anotada)

S3237 ¿Toma estabilizadores de diuresis?

○ Yes
○ No
((Betmiga, unibenestan))

S32371 Detalle los estabilizadores de diuresis con posología

____
(De la lista anotada)

S3238 ¿Toma sedantes o inductores de sueño?

○ Yes
○ No
((Lorazepam, alprazolam, quetiapina))

S32381 Detalle los sedantes / inductores de sueño con posología

____
(De la lista anotada)

S3239 ¿Toma antipruriginosos?

○ Yes
○ No
((Cetirizina, desclorfeniramina))

S32391 Detalle los antipruriginosos con posología

____
(De la lista anotada)

S32310 ¿Toma ansiolíticos, calmantes o moduladores de afecto?

○ Yes
○ No
((Diazepam, fenitoína, carbamazepina, haloperidol))

S323101 Detalle los ansiolíticos / calmantes / moduladores de afecto con posología

____
(De la lista anotada)

S32311 ¿Toma medicamentos para la depresión?

○ Yes
○ No
((Sertralina, fluoxetina, duloxetina))

S323111 Detalle los anti-depresivos con posología

____
(De la lista anotada)

S32312 ¿Toma medicamentos para la disnea / "falta de aire"?

○ Yes
○ No
((Morfina, oxígeno))

S323121 Detalle los medicamentos para la disnea / "falta de aire" con posología

____
(De la lista anotada)

S32313 ¿Toma medicamentos mucolíticos?

◯ Yes
◯ No
((N-acetil-cisteína))

S323131 Detalle los medicamentos mucolíticos con posología

____
(De la lista anotada)

S32314 ¿Toma medicamentos para mejorar/ aumentar el apetito / nutrir?

◯ Yes
◯ No
((Megestrol, batidos proteínas))

S323141 Detalle los medicamentos mejorar/aumentar el apetito/nutrir con posología

____
(De la lista anotada)

S32315 ¿Toma medicamentos diuréticos?

◯ Yes
◯ No
((Furosemida, HCTZ, Espironolactona))

S323151 Detalle los medicamentos diuréticos con posología

____
(De la lista anotada)

S32316 ¿Toma medicamentos inhalados?

◯ Yes
◯ No
((Terbutalina, atrovent, salmeterol, corticoides))

S323161 Detalle los medicamentos inhalados con posología

____
(De la lista anotada)

S32317 ¿Toma medicamentos vasodilatadores coronarios / antianginosos?

◯ Yes
◯ No
((Cafinitrina, solinitrina, nitroderm, nitrodur))

S323171 Detalle los medicamentos vasodilatadores coronarios / antianginosos con posología

____
(De la lista anotada)

S32318 ¿Toma medicamentos antiagregantes plaquetarios?

◯ Yes
◯ No
((AAS, clopidogrel, prasugrel, ticagrelor))

S323181 Detalle los medicamentos antiagregantes plaquetarios con posología

____
(De la lista anotada)

S32319 ¿Toma medicamentos anticoagulantes orales / inyectados?	◯ Yes<br>◯ No<br>((Dabigatrán, Rivaroxabán, Apixabán, Edoxabán, Acenocumarol, Warfarina))
S323191 Detalle los medicamentos anticoagulantes orales / inyectados con posología	____________________<br>(De la lista anotada)
S32320 ¿Toma medicamentos anti-hipertensivos?	◯ Yes<br>◯ No<br>((Losartán, Valsartán, Irbesaltán, Amlodipino, Enalapril))
S323201 Detalle los medicamentos anti-hipertensivos con posología	____________________<br>(De la lista anotada)
S32321 ¿Toma medicamentos anti-arrítmicos?	◯ Yes<br>◯ No<br>((Bisoprolol, amiodarona, digitálicos, beta-bloqueadores, antagonistas canales calcio, bloqueantes canales sodio))
S323211 Detalle los medicamentos anti-arrítmicos con posología	____________________<br>(De la lista anotada)
S32322 ¿Toma medicamentos hipoglicemiantes?	◯ Yes<br>◯ No<br>((Metformina, jardiance, insulina rápida / lenta))
S323221 Detalle los medicamentos hipoglicemiantes con posología	____________________<br>(De la lista anotada)
S32323 ¿Toma medicamentos para otras enfermedades (diferente a la que indicó el CP)?	◯ Yes<br>◯ No<br>(Otras enfermedades diferentes a las que indican el CP)
S323231 Detalle los medicamentos para otras enfermedades/condiciones (NO la que motiva el CP)	____________________
S324 ¿Ha utilizado transporte medicalizado?	◯ Yes<br>◯ No<br>((Ambulancia, furgoneta))
S325 Número de veces (aproximado) de uso transporte medicalizado	____________________

S426 En el último mes, ¿ha pagado por alguno de estos conceptos por causa del CP?; en caso de haber pagado, ¿cuánto ha sido?	
S4261 Servicios privados de salud (mutua)	◯ Yes<br>◯ No<br>((Mutua))
S42611 Pago aproximado servicios privados salud (mutua)	__________________________________
S4262 Ayudas para caminar	◯ Yes<br>◯ No<br>((Bastón, muleta, silla de ruedas, scooter))
S42621 Pago aproximado por ayudas para caminar	__________________________________
S4263 Ayudas de comunicación, sistemas aumentativos, tele-asistencia, sistemas telefónicos	◯ Yes<br>◯ No<br>((Tablet, audífonos, gafas, botón de emergencia/SOS))
S42631 Pago aproximado por ayudas de comunicación, sistemas aumentativos, tele-asistencia, sistemas telefónicos	__________________________________
S4264 Ayudas respiratorias	◯ Yes<br>◯ No<br>((O2 en casa, respirador no invasivo, CPAP))
S42641 Pago aproximado por ayudas respiratorias	__________________________________
S4265 Elementos de casa	◯ Yes<br>◯ No<br>((Ropa, sábanas, almohadas, protectores almohadas /colchón))
S42651 Pago aproximado por elementos de casa	__________________________________
S4266 Ayuda por/para la incontinencia	◯ Yes<br>◯ No<br>((Compresas, pañales, sábanas, impermeables, cobertores sillas))
S42661 Pago aproximado ayudas por/para incontinencia	__________________________________
S4267 Modificaciones arquitectónicas en casa	◯ Yes<br>◯ No<br>((Rampas, rieles, ascensores, extensiones de casa))

S42671 Pago aproximado modificaciones arquitectónicas en casa	__________________________________
S4268 Cuidado profesional sanitario EN CASA	◯ Yes<br>◯ No<br>((Terapia ocupacional, psicólogo, trabajo social, nutricionista))
S42681 Pago aproximado cuidado profesional sanitario EN CASA	__________________________________
S4269 Cuidado profesional sanitario FUERA DE CASA	◯ Yes<br>◯ No<br>((Centro de día))
S42691 Pago aproximado cuidado profesional sanitario EN CASA	__________________________________
S42610 Cuidado profesional PRIVADO EN CASA	◯ Yes<br>◯ No<br>((Auxiliar, acompañante))
S426101 Pago aproximado cuidado profesional PRIVADO EN CASA	__________________________________
S42611* Cuidado por familiar y/o amigo	◯ Yes<br>◯ No<br>((Familiar, amigo))
S426111* Pago aproximado cuidado por familiar / amigo	__________________________________
S42612 Cuidados espirituales	◯ Yes<br>◯ No<br>((Sacerdote, rabino, maestro, guía espiritual))
S426121 Pago aproximado por cuidados espirituales	__________________________________
S42613 Adaptaciones en auto	◯ Yes<br>◯ No<br>((Rampa para silla de ruedas, cambio de auto))
S426131 Pago aproximado por adaptaciones en auto	__________________________________
S42614 Terapias alternativas /medicina integrativa	◯ Yes<br>◯ No<br>((Acupuntura, aromaterapia, moxibustión, quiropraxia))

S426141 Pago aproximado por terapias alternativas / medicina integrativa	__________________
S42615 Medicación alternativa / integrativa	◯ Yes<br>◯ No<br>((Medicamentos homeopáticos, chinos, ayurvédicos))
S426151 Pago aproximado por medicinas alternativas / integrativas	__________________

S427 ¿Ha utilizado algún medio de transporte o aparcamiento pagado por usted EN EL ÚLTIMO MES, relacionado con el CP?

S4271 Taxi	◯ Yes<br>◯ No
S42711 Número aproximado de viajes en TAXI	__________________
S427111 Coste aproximado de CADA viaje en TAXI	__________________
S4272 Bus	◯ Yes<br>◯ No
S42721 Número aproximado de viajes en BUS	__________________
S427211 Coste aproximado de CADA viaje en BUS	__________________
S4273 Coche particular	◯ Yes<br>◯ No
S42731 Número aproximado de usos de COCHE PARTICULAR	__________________
S427311 Coste aproximado de CADA uso de COCHE PARTICULAR	__________________
S4274 Parquing	◯ Yes<br>◯ No
S42741 Número aproximado de usos de PARQUING	__________________
S427411 Coste aproximado de CADA uso de PARQUING	__________________
S428 ¿Ha tenido que comprar medicación de CP SIN RECETA, en el ÚLTIMO MES?	◯ Yes<br>◯ No

S4281 Nombre Medicamento1 / Coste Medicamento1

____
(Anotar Nombre y Coste en Euros)

S4282 Nombre Medicamento2 / Coste Medicamento2

____
(Anotar Nombre y Coste en Euros)

S4283 Nombre Medicamento3 / Coste Medicamento3

____
(Anotar Nombre y Coste en Euros)

S5 Costes relacionados con el tiempo de trabajo perdido del paciente

S529 Si es empleado o trabaja por cuenta propia, ¿ha dejado de trabajar o ha perdido productividad debido a su enfermedad/diagnóstico en su trabajo (incluyendo asistir a citas médicas)?

◯ Yes
◯ No

S5291 Absentismo laboral (pérdida productividad en trabajo)

◯ Yes
◯ No

S52911 Número días absentismo

____
(Anotar número de días.)

S52912 Importe absentismo (€)

____
(Importe en Euros.)

S5292 Baja temporal (incapacidad temporal)

◯ Yes
◯ No
(Entre 1 - 1.5 años)

S52921 Número días baja temporal

____
(Anotar número de días.)

S52922 Importe baja temporal (€)

____
(Anotar importe en Euros.)

S5293 Baja larga duración (incapacidad permanente)

◯ Yes
◯ No
(Más de 1 -1.5 años)

S52931 Número días baja larga

____
(Anotar número de días.)

S52932 Importe baja larga (€)

____
(Anotar importe en Euros)

S5294 Jubilación anticipada

◯ Yes
◯ No

S52941 Años jubilación

____
(Anotar número de años.)

S52942 Importe Jubilación (€)

____
(Anotar importe en Euros.)

S5295 Pérdida Tiempo Ocio

◯ Yes
◯ No

S52951 Días de Ocio

____
(Anote número de días.)

S52952 Importe Días de Ocio (€)

____
(Anotar importe en Euros.)

S6 Datos Clínicos / Índices

S631 Índice Charlson

◯ Yes
◯ No

Charlson EDAD_6 (< 50 años=0 / 50-59 años=1 / 60-69 años=2 / 70-79 años=3 / >=80 años=4)

____

Charlson IAM_1 (Sí ["Así es"] = 1 / No =0)

____

Charlson ICC_1 (Sí ["Así es"] = 1 / No =0)

____

Charlson AEOP_1 (Sí ["Así es"] = 1 / No =0)

____

Charlson ECV_1 (Sí ["Así es"] = 1 / No =0)

____

Charlson Demencia_1 (Sí ["Así es"] = 1 / No =0)

____

Charlson EPOC_1 (Sí ["Así es"] = 1 / No =0)

____

Charlson T Conectivo_1 (Sí ["Así es"] = 1 / No =0)

____

Charlson U GDuodeno_1 (Sí ["Así es"] = 1 / No =0)

____

Charlson Hepato Leve_1 (Sí ["Así es"] = 1 / No =0)

____

Charlson DM No Complicaciones_1 (Sí ["Así es"] = 1 / No =0)

____

Charlson Hemi/Paraplejia_2 (Sí ["Así es"] = 2 / No =0) ________________________________

Charlson IRC_2 (Sí ["Así es"] = 2 / No =0) ________________________________

Charlson DM Lesión_2 (Sí ["Así es"] = 2 / No =0) ________________________________

Charlson Neoplasia sólida_6 (Ninguna=0 / Localizada=2 / Metástasis=6) ________________________________

Charlson Leucemia_2 (Sí ["Así es"] = 2 / No =0) ________________________________

Charlson Linfoma_2 (Sí ["Así es"] = 2 / No =0) ________________________________

Charlson Hepato Moderada/Severa_3
(Sí ["Así es"] = 3 / No =0) ________________________________

Charlson SIDA_6
(Sí ["Así es"] = 6 / No =0) ________________________________

Sumatoria Charlson Auto ________________________________

S6311 Detalle Índice Charlson

S63111 Sumatoria Índice Charlson ________________________________
(Anote Sumatoria Charlson y % sobrevida a 10 años)

S632 Criterios Fenotipo Fragilidad (FRAIL)

S6321 (F) Fatiga
¿Está usted fatigado?
(Sí ["Así es"] = 1 / No =0) ________________________________

S6322 (R) Resistencia
¿No puede caminar un tramo de escaleras?
(Sí ["Así es"] = 1 / No =0) ________________________________

S6323 (A) Aeróbico
¿No puede caminar una manzana?
(Sí ["Así es"] = 1 / No =0) ________________________________

S6324 (I) Enfermedades
¿Tiene más de 5 enfermedades?
(Sí ["Así es"] = 1 / No =0) ________________________________

S6325 (L) Pérdida peso
¿Ha perdido más del 5% de su peso en los pasados 6 meses?
(Sí ["Así es"] = 1 / No =0) ________________________________

S6326 Sumatoria FRAIL

____
(Sumatoria FRAIL (=>3: Frailty; 1-2: Pre-frail))

Índice Barthel (Dependencia ABVD)

S633 Índice Barthel (Independencia ABVD)
◯ Yes
◯ No

Barthel Comer
Totalmente independiente-10
Necesita ayuda para cortar carne, el pan, etc.-5
Dependiente-0

____

Barthel Lavarse
Independiente: entra y sale solo del baño-5
Dependiente-0

____

Barthel Vestirse
Independiente: capaz de ponerse y de quitarse la ropa, abotonarse, atarse los zapatos-10
Necesita ayuda-5
Dependiente-0

____

Barthel Arreglarse
Independiente para lavarse la cara, las manos, peinarse, afeitarse, maquillarse, etc.-5
Dependiente-0

____

Barthel Deposición
10, Continencia normal-10
5, Ocasionalmente algún episodio de incontinencia, o necesita ayuda para administrarse supositorios o lavativas-5
0, Incontinencia -0

____
((Valórese semana previa))

Barthel Micción
Continencia normal, o es capaz de cuidarse de la sonda si tiene una puesta -10
Un episodio diario como máximo de incontinencia, o necesita ayuda para cuidar de la sonda -5
Incontinencia -0

____
((Valórese semana previa))

Barthel Retrete
Independiente para ir al cuarto de aseo, quitarse y ponerse la ropa...-10
Necesita ayuda para ir al retrete, pero se limpia solo -5
Dependiente -0

____

Barthel Trasladar
Independiente para ir del sillón a la cama-15
Mínima ayuda física o supervisión para hacerlo -10
Necesita gran ayuda, pero es capaz de mantenerse sentado solo -5
Dependiente -0

____

Barthel Deambular
Independiente, camina solo 50 metros -15
Necesita ayuda física o supervisión para caminar 50 metros-10
Independiente en silla de ruedas sin ayuda -5
Dependiente -0

____

Barthel Escalones
Independiente para bajar y subir escaleras -10
Necesita ayuda física o supervisión para hacerlo -5
Dependiente -0

____

S63311 Sumatoria Barthel Automática

____
(Anote Sumatoria Barthel (___ /100). Dependencia: < 20 Total; 21-60 Grave; 61-90 Moderada; 91-99 Leve; 100 Independiente)

S6331 Detalle Índice Barthel

S634 Escala De Jong Gierveld (Valoración soledad)

S6341 Siempre hay alguien con quien puedo hablar sobre mis problemas del día a día.
◯ No
◯ Más o menos
◯ Sí

S6342 Extraño tener un amigo cercano
◯ No
◯ Más o menos
◯ Sí

S6343 Experimento una sensación general de soledad.
◯ No
◯ Más o menos
◯ Sí

S6344 Hay muchas personas en las cuales me puedo apoyar cuando tengo problemas.
◯ No
◯ Más o menos
◯ Sí

S6345 Extraño en placer de la compañía de otros.
◯ No
◯ Más o menos
◯ Sí

S6346 Encuentro que mi círculo de amigos y conocidos es muy limitado
◯ No
◯ Más o menos
◯ Sí

S6347 Hay muchas personas en las que puedo confiar por completo.
◯ No
◯ Más o menos
◯ Sí

S6348 Hay suficiente gente a la que me siento cercana.
◯ No
◯ Más o menos
◯ Sí

S6349 Extraño tener personas alrededor mío
◯ No ◯ Más o menos
◯ Sí

S63410 Con frecuencia me siento rechazado.	◯ No ◯ Más o menos<br>◯ Sí
S63411 Puedo llamar a mis amigos siempre que los necesite.	◯ No ◯ Más o menos<br>◯ Sí
S634121 Sumatoria Emotional Loneliness, De Jong Gierveld	______________________
S634122 MISSING Emotional Loneliness, De Jong Gierveld	______________________<br>((Sumatoria))
S634123 Sumatoria Social Loneliness De Jong Gierveld	______________________
S634124 MISSING Social Loneliness De Jong Gierveld	______________________<br>((Sumatoria))
S63412_ TOTAL LONELINESS SCORE	______________________
S63413 Interpretación Escala De Jong Gierveld	◯ No soledad (puntaje 0, 1 o 2)<br>◯ Soledad moderada (puntaje 3-8)<br>◯ Soledad severa (9-10)<br>◯ Soledad MUY severa (11)<br>(Revisar resultado de Missing emotional loneliness score / Missing social loneliness score para determinar validez del resultado (cada uno debería ser 0))

S735 EQ5D5L PACIENTE. © EuroQol Research Foundation. EQ-5D™ is a trade mark of the EuroQol Research Foundation.

S7351 Haga clic en UNA casilla, la que mejor describe su MOVILIDAD en el día de HOY.	◯ No tengo problemas para caminar-1<br>◯ Tengo problemas leves para caminar-2<br>◯ Tengo problemas moderados para caminar-3<br>◯ Tengo problemas graves para caminar-4<br>◯ No puedo caminar-5
S73511 Resultado Movilidad	______________________
S7352 Haga clic en UNA casilla, la que mejor describe su AUTO-CUIDADO en el día de HOY.	◯ No tengo problemas para lavarme o vestirme-1<br>◯ Tengo problemas leves para lavarme o vestirme-2<br>◯ Tengo problemas moderados para lavarme o vestirme-3<br>◯ Tengo problemas graves para lavarme o vestirme-4<br>◯ No puedo lavarme o vestirme-5
S73521 Resultado Auto-Cuidado	______________________

S7353 Haga clic en UNA casilla, la que mejor describe su capacidad para ACTIVIDADES COTIDIANAS en el día de HOY.	◯ No tengo problemas para realizar mis actividades cotidianas-1<br>◯ Tengo problemas leves para realizar mis actividades cotidianas-2<br>◯ Tengo problemas moderados para realizar mis actividades cotidianas-3<br>◯ Tengo problemas graves para realizar mis actividades cotidianas-4<br>◯ No puedo realizar mis actividades cotidianas-5
S73531 Resultado Actividades Cotidianas	______________________
S7354 Haga clic en UNA casilla, la que mejor describe su DOLOR/MALESTAR en el día de HOY.	◯ No tengo dolor ni malestar-1<br>◯ Tengo dolor o malestar leve-2<br>◯ Tengo dolor o malestar moderado-3<br>◯ Tengo dolor o malestar fuerte-4<br>◯ Tengo dolor o malestar extremo-5
S73541 Resultado Dolor/Malestar	______________________
S7355 Haga clic en UNA casilla, la que mejor describe su ANSIEDAD/DEPRESIÓN en el día de HOY.	◯ No estoy ansioso ni deprimido-1<br>◯ Estoy levemente ansioso o deprimido-2<br>◯ Estoy moderadamente ansioso o deprimido-3<br>◯ Estoy muy ansioso o deprimido-4<br>◯ Estoy extremadamente ansioso o deprimido-5
S73551 Resultado Ansiedad/Depresión	______________________
S7366 Estado de salud (España)	______________________<br>(Anote la secuencia de números resultante de las cinco (5) respuestas: S73511 /S73521 /S73531 /S73541 /S73551.)
S7367 Nos gustaría conocer lo buena o mala que es su salud HOY.<br>La escala está numerada del 0 al 100.<br>100 representa la MEJOR salud que usted se pueda imaginar.<br>0 representa la PEOR salud que usted se pueda imaginar.<br>Haga clic en la escala para indicar cuál es su estado de salud HOY.	0 50 100<br>*(Place a mark on the scale above)*

S2_Cuestionario_Cuidador_Spanish

Por favor completa TODOS LOS DATOS que se solicitan a continuación.

¡Gracias! :)

Digitador

◯ AHPB
◯ CD
◯ MTB
◯ JPE
(Iniciales del digitador de los datos)

Centro_2

◯ Clínic
◯ Mollet
◯ Granollers
◯ Otro
(Se trata del Centro de referencia del Paciente.)

Detalle Otro Centro_2

____
(Si es Otro, por favor detallar)

ID Cuidador_2 (¡Atención!)*

____
(*¡Anote el número de H. Clinica del Paciente, seguido de "_2"!)

Nombre Cuidador

____

S1 Datos Socio-Demográficos

S11 Fecha Entrevista Cuidador

____

S12 Género Cuidador

◯ Femenino
◯ Masculino
◯ Otros

S13 Si la respuesta es otros, especificar

____

S14 Fecha nacimiento Cuidador

____

S15 Código Postal Residencia Cuidador

____

S16 País Origen Cuidador

◯ España
◯ Extranjero

S17 Si es Extranjero

◯ Europa norte
◯ Europa centro
◯ Europa este
◯ Europa sur
◯ Resto Europa
◯ Reino Unido / Irlanda
◯ África
◯ Asia / Australia / Oceanía
◯ América Norte
◯ América Centro
◯ América Sur

S18 Estado Civil Cuidador

◯ Soltero/a
◯ Casado/a
◯ Divorciado/a - Separado/a
◯ Viudo/a
◯ Desconocido

S19 ¿Usted vide con la persona que cuida?

◯ Yes
◯ No

S110 Número de personas que habitan en vivienda del Cuidador

____

S111 Nivel de estudios Cuidador

◯ No sabe leer o escribir
◯ Educación primaria incompleta
◯ Educación Primaria completa
◯ Primera etapa de enseñanza secundaria, con o sin título
◯ Estudios de Bachillerato
◯ Enseñanzas profesionales de grado medio o equivalente
◯ Enseñanzas profesionales de grado superior o equivalente
◯ Estudios universitarios o equivalente
◯ Desconocido

S112 Situación laboral actual Cuidador

◯ Trabaja por cuenta ajena
◯ Trabaja por cuenta propia
◯ Jubilado o pensionista (anteriormente ha trabajado)
◯ Parado/a y ha trabajado antes
◯ Parado/a y busca su primer empleo
◯ Estudiante
◯ Incapacitado/a para trabajar
◯ Trabajo doméstico no remunerado
◯ Otra situación
◯ Desconocido

S113 Si trabaja por cuenta ajena, seleccione la categoría laboral en la cual se encuentra el Cuidador	◯ Directores y gerentes<br>◯ Técnicos y profesionales científicos e intelectuales de la salud y la enseñanza<br>◯ Otros técnicos y profesionales científicos e intelectuales<br>◯ Técnicos; profesionales de apoyo<br>◯ Empleados de oficina que no atienden al público<br>◯ Empleados de oficina que atienden al público<br>◯ Trabajadores de los servicios de restauración y comercio<br>◯ Trabajadores de los servicios de salud y el cuidado de las personas<br>◯ Trabajadores de los servicios de protección y seguridad<br>◯ Trabajadores cualificados en el sector agrícola, ganadero, forestal y pesquero<br>◯ Trabajadores cualificados de la construcción, excepto operadores de máquinas<br>◯ Trabajadores cualificados de las industrias manufactureras, excepto operadores de instalaciones y máquinas<br>◯ Operadores de instalaciones y máquinas fijas, y monitores<br>◯ Conductores y operadores de maquinaria móvil<br>◯ Trabajadores no cualificados en servicios (excepto transportes)<br>◯ Peones de la agricultura, pesca, construcción, industrias manufactureras y transportes<br>◯ Ocupaciones militares<br>◯ Desconocido
S114 Especifique la profesión del Cuidador, si no puede clasificarla en la entrevista, en las categorías anteriores.	__________________________________<br>(PARA EL ENTREVISTADOR)
S115 Disponibilidad de un plan privado de salud del Cuidador	◯ Sí<br>◯ No<br>◯ NS/NC
S116 ¿Cuál es el importe mensual neto de los ingresos totales del hogar del Cuidador en la actualidad?	◯ Menos de 500 €<br>◯ De 500 € a menos de 1.000 €<br>◯ De 1.000 € a menos de 1.500 €<br>◯ De 1.500 € a menos de 2.000 €<br>◯ De 2.000 € a menos de 2.500 €<br>◯ De 2.500 € a menos de 3.000 €<br>◯ De 3.000 € a menos de 5.000 €<br>◯ De 5.000 € a menos de 7.000 €<br>◯ De 7.000 € a menos de 9.000 €<br>◯ 9.000 € y más €<br>((ingresos percibidos por todos los miembros del hogar, aporten o no estos ingresos o parte de los mismos para sufragar los gastos del hogar))
S117 ¿Cuál es su relación con el Paciente?	◯ Es mi pareja/ marido/ mujer/ compañero/a<br>◯ Es mi madre/ padre<br>◯ Es mi hermana/ o<br>◯ Es mi hija/ o<br>◯ Otro
S1171 Si es la respuesta es Otro, especifique	__________________________________

S2 Sobre los Cuidados Paliativos (Cuidador)

S218 ¿Cuál es el diagnostico principal por el que inició el programa de cuidados paliativos la persona que usted cuida?

- ◯ Diagnóstico oncológico
- ◯ Diagnostico respiratorio
- ◯ Diagnostico cardiovascular
- ◯ Otros

S2181 Si la respuesta es otros, especificar

____

S219 ¿Desde qué fecha le indicaron al Paciente los cuidados paliativos?

____

S2191 ¿Desde qué fecha cuida usted al Paciente?

____

S2192 Tiempo como Cuidador (en meses)

____

S220 ¿Cuántas HORAS dedica usted COMO MEDIA y EN UN DÍA NORMAL al cuidado del paciente?

____

S221 ¿Cuántos DÍAS, EN DÍAS NORMALES, dedica usted COMO MEDIA al cuidado del paciente?

____

S222 Durante el último mes de cuidado del paciente, ¿cuánto tiempo ha dedicado AL DÍA, en un DÍA NORMAL, en las siguientes actividades?

S2221 Cuidado personal básico (aseo, vestido)

____
(Anote las horas/día)

S2222 Movilización (caminar, silla de ruedas)

____
(Anote las horas/día)

S2223 Asistencia para PREPARAR alimentos

____
(Anote las horas/día)

S2224 Asistencia para CONSUMIR alimentos

____
(Anote las horas/día)

S2225 Asistencia en tareas domésticas (lavar ropa, limpiar, hacer compra)

____
(Anote las horas/día)

S2226 Asistencia para organizar/ administrar medicamentos (pastillero, orales, inyectados)

____
(Anote las horas/día)

S2227 Asistencia para realizar curas

____
(Anote las horas/día)

S2228 Vigilancia y supervisión

____
(Anote las horas/día)

S2229 Programar visitas y llevar/traer de visitas

____
(Anote las horas/día)

S22210 Recoger medicación en hospital/ farmacia / organizar pastillero

____
(Anote las horas/día)

S22211 Manejar finanzas o hacer papeleo

____
(Anote las horas/día)

S22212 Compartir tiempo juntos/ con la familia

____
(Anote las horas/día)

S22213 Otros

____
(Anote las horas/día)

Sumatoria Tiempo Cuidador

____

Detalle actividades OTROS CUIDADORES NO PROFESIONALES

S223 ¿Tiene el Paciente otros cuidadores (familiares / no profesionales)?

- ◯ Yes
- ◯ No

S2231 ¿Cuál es la relación del paciente con esos otros cuidadores NO PROFESIONALES?

- ◯ Pareja / marido /mujer /compañera/o
- ◯ Hija/o
- ◯ Hermana /o
- ◯ Nuera/yerno
- ◯ Otros

S2232 ¿Cuántas horas dedican otros cuidadores NO PROFESIONALES, como media y EN UN DÍA NORMAL, al cuidado del Paciente?

____

S2233 ¿Cuántos DÍAS A LA SEMANA dedican otros cuidadores NO PROFESIONALES, como media al cuidado del Paciente?

____

S2234 Durante el ÚLTIMO MES de cuidado, ¿cuánto tiempo AL DÍA ha dedicado el CUIDADOR NO PROFESIONAL, en un DÍA NORMAL, en las siguientes actividades?

S22341 Cuidado personal básico (aseo, vestido)

____
(Anote las horas/día)

S22342 Movilización (caminar, silla de ruedas)

____
(Anote las horas/día)

S22346 Asistencia para organizar/ administrar medicamentos (pastillero, orales, inyectados)	________________ (Anote las horas/día)
S22343 Asistencia para PREPARAR alimentos	________________ (Anote las horas/día)
S22344 Asistencia para CONSUMIR alimentos	________________ (Anote las horas/día)
S22345 Asistencia en tareas domésticas (lavar ropa, limpiar, hacer compra)	________________ (Anote las horas/día)
S22347 Asistencia para realizar curas	________________ (Anote las horas/día)
S22348 Vigilancia y supervisión	________________ (Anote las horas/día)
S22349 Programar visitas y llevar/traer de visitas	________________ (Anote las horas/día)
S223410 Recoger medicación en hospital/ farmacia / organizar pastillero	________________ (Anote las horas/día)
S223411 Manejar finanzas o hacer papeleo	________________ (Anote las horas/día)
S223412 Compartir tiempo juntos/ con la familia	________________ (Anote las horas/día)
S223413 Otros	________________ (Anote las horas/día)
Sumatoria Tiempo Cuidador (No Profesional)	________________

S3 Costes Directos No Sanitarios para el Cuidador.

En el ÚLTIMO MES, en su rol de Cuidador, ¿ha pagado por alguno de los siguientes conceptos?

S3211 Servicios privados de salud (mutua)	◯ Yes<br>◯ No<br>((Mutua))
S32111 Pago aproximado servicios privados salud (mutua) (€)	__________________
S3212 Ayudas para caminar	◯ Yes<br>◯ No<br>((Bastón, muleta, silla de ruedas, scooter))
S32121 Pago aproximado por ayudas para caminar (€)	__________________
S3213 Ayudas de comunicación, sistemas aumentativos, tele-asistencia, sistemas telefónicos	◯ Yes<br>◯ No<br>((Tablet, audífonos, gafas, botón de emergencia/SOS))
S32131 Pago aproximado por ayudas de comunicación, sistemas aumentativos, tele-asistencia, sistemas telefónicos (€)	__________________
S3214 Ayudas respiratorias	◯ Yes<br>◯ No<br>((O2 en casa, respirador no invasivo, CPAP))
S32141 Pago aproximado por ayudas respiratorias (€)	__________________
S3215 Elementos de casa	◯ Yes<br>◯ No<br>((Ropa, sábanas, almohadas, protectores almohadas /colchón))
S32151 Pago aproximado por elementos de casa (€)	__________________
S3216 Ayuda por/para la incontinencia	◯ Yes<br>◯ No<br>((Compresas, pañales, sábanas, impermeables, cobertores sillas))
S32161 Pago aproximado ayudas por/para incontinencia (€)	__________________
S3217 Modificaciones arquitectónicas en casa	◯ Yes<br>◯ No<br>((Rampas, rieles, ascensores, extensiones de casa, plato de ducha))
S32171 Pago aproximado modificaciones arquitectónicas en casa (€)	__________________

S3218 Cuidado profesional sanitario EN CASA	◯ Yes<br>◯ No<br>((Terapia ocupacional, psicólogo, trabajo social, nutricionista))
S32181 Pago aproximado cuidado profesional sanitario EN CASA (€)	__________________________
S3219 Cuidado profesional sanitario FUERA DE CASA	◯ Yes<br>◯ No<br>((Centro de día))
S32191 Pago aproximado cuidado profesional sanitario FUERA DE CASA (€)	__________________________
S32110 Cuidado profesional PRIVADO EN CASA	◯ Yes<br>◯ No<br>((Auxiliar, acompañante))
S321101 Pago aproximado cuidado profesional PRIVADO EN CASA (€)	__________________________
S32111 Cuidado por familiar y/o amigo	◯ Yes<br>◯ No<br>((Familiar, amigo))
S321111 Pago aproximado cuidado por familiar / amigo (€)	__________________________
S32112 Cuidados espirituales	◯ Yes<br>◯ No<br>((Sacerdote, rabino, maestro, guía espiritual))
S321121 Pago aproximado por cuidados espirituales (€)	__________________________
S32113 Adaptaciones en auto	◯ Yes<br>◯ No<br>((Rampa para silla de ruedas, cambio de auto))
S321131 Pago aproximado por adaptaciones en auto (€)	__________________________
S32114 Terapias alternativas /medicina integrativa	◯ Yes<br>◯ No<br>((Acupuntura, aromaterapia, moxibustión, quiropraxia))
S321141 Pago aproximado por terapias alternativas / medicina integrativa (€)	__________________________

S32* ¿Ha utilizado algún medio de transporte o aparcamiento pagado por usted, relacionado con el CP, EN EL ÚLTIMO MES?

S32115 Taxi
○ Yes
○ No

S321151 Número aproximado de viajes en TAXI
____

S3211511 Coste aproximado de CADA viaje en TAXI (€)
____

S32116 Bus
○ Yes
○ No

S321161 Número aproximado de viajes en BUS
____

S3211611 Coste aproximado de CADA viaje en BUS (€)
____

S32117 Coche particular
○ Yes
○ No

S321171 Número aproximado de usos de COCHE PARTICULAR
____

S3211711 Coste aproximado de CADA uso de COCHE PARTICULAR (€)
____

S32118 Parquing
○ Yes
○ No

S321181 Número aproximado de usos de PARQUING
____

S3211811 Coste aproximado de CADA uso de PARQUING (€)
____

S4 Costes relacionados con el tiempo de trabajo perdido del Cuidador

S422 Si es empleado o trabaja por cuenta propia, ¿ha dejado de trabajar o ha perdido productividad debido a su actividad como Cuidador? (incluyendo acompañar a citas médicas)?
○ Yes
○ No

S4221 Absentismo laboral (pérdida productividad en trabajo)
○ Yes
○ No

S42211 Número días absentismo
____
(Anotar número de días.)

S42212 Importe absentismo (€)
____
(Anotar importe en Euros.)

S4222 Baja temporal	◯ Yes<br>◯ No
S42221 Número días baja temporal	________________<br>(Anotar número de días.)
S42222 Importe baja temporal (€)	________________<br>(Anotar importe en Euros.)
S4223 Baja larga duración	◯ Yes<br>◯ No
S42231 Número días baja larga	________________<br>(Anotar número de días.)
S42232 Importe baja larga (€)	________________<br>(Anotar importe en Euros.)
S4224 Jubilación anticipada	◯ Yes<br>◯ No
S42241 Años jubilación anticipada	________________<br>(Anotar años.)
S42242 Importe Jubilación (€)	________________<br>(Anotar importe en Euros.)
S4225 Tiempo cuidados médicos	◯ Yes<br>◯ No
S42251 Días Cuidados Médicos	________________<br>(Anotar número de días.)
S42252 Importe Días de Cuidados Médicos	________________<br>(Anotar importe en Euros.)
S4226 Pérdida Tiempo Ocio	◯ Yes<br>◯ No
S42261 Días de Ocio	________________<br>(Anote número de días.)
S42262 Importe Días de Ocio (€)	________________<br>(Anote importe en Euros.)
S423 ¿Usted cobra por cuidar al Paciente?	◯ Yes<br>◯ No

S4231 Dado que cobra, ¿cuál es el importe mensual por su rol de cuidador? (€) __________________________________

S5 Estado de Salud del Cuidador

S524 En el último mes, ¿ha tenido gastos relacionados con problemas de salud suyos, y que sean resultado de su rol de cuidador?
- ◯ Yes
- ◯ No

S52411 Concepto Gasto_1 __________________________________

S524111 Coste Gasto_1 (€) __________________________________

S52412 Concepto Gasto_2 __________________________________

S524121 Coste Gasto_2 (€) __________________________________

S52413 Concepto Gasto_3 __________________________________

S524131 Coste Gasto_3 (€) __________________________________

S525 Escala Sobrecarga Zarit (modified short-form) (todas las preguntas anotadas a continuación deberán ser respondidas con alguna opción, POR FAVOR MARQUE SOLAMENTE UNA DE LAS OPCIONES

S5251 ¿Siente que su familiar solicita más ayuda de la que realmente necesita?
- ◯ Nunca
- ◯ Casi nunca
- ◯ A veces
- ◯ Frecuentemente
- ◯ Casi siempre

S5252 ¿Siente que debido al tiempo que dedica a su familiar ya no dispone de tiempo suficiente para usted?
- ◯ Nunca
- ◯ Casi nunca
- ◯ A veces
- ◯ Frecuentemente
- ◯ Casi siempre

S5253 ¿Se siente tenso cuando tiene que cuidar a su familiar y atender además otras responsabilidades?
- ◯ Nunca
- ◯ Casi nunca
- ◯ A veces
- ◯ Frecuentemente
- ◯ Casi siempre

S5254 ¿Se siente avergonzado por la conducta de su familiar?
- ◯ Nunca
- ◯ Casi nunca
- ◯ A veces
- ◯ Frecuentemente
- ◯ Casi siempre

S5255 ¿Se siente enfadado cuando está cerca de su familiar?	◯ Nunca<br>◯ Casi nunca<br>◯ A veces<br>◯ Frecuentemente<br>◯ Casi siempre
S5256 ¿Cree que la situación actual afecta de manera negativa a su relación con amigos y otros miembros de su familia?	◯ Nunca<br>◯ Casi nunca<br>◯ A veces<br>◯ Frecuentemente<br>◯ Casi siempre
S5257 ¿Siente temor por el futuro que le espera a su familiar?	◯ Nunca<br>◯ Casi nunca<br>◯ A veces<br>◯ Frecuentemente<br>◯ Casi siempre
S5258 ¿Siente que su familiar depende de usted?	◯ Nunca<br>◯ Casi nunca<br>◯ A veces<br>◯ Frecuentemente<br>◯ Casi siempre
S5259 ¿Se siente agobiado cuando tiene que estar junto a su familiar?	◯ Nunca<br>◯ Casi nunca<br>◯ A veces<br>◯ Frecuentemente<br>◯ Casi siempre
S52510 ¿Siente que su salud se ha resentido por cuidar a su familiar?	◯ Nunca<br>◯ Casi nunca<br>◯ A veces<br>◯ Frecuentemente<br>◯ Casi siempre
S52511 ¿Siente que no tiene la vida privada que desearía debido a su familiar?	◯ Nunca<br>◯ Casi nunca<br>◯ A veces<br>◯ Frecuentemente<br>◯ Casi siempre
S52512 ¿Cree que su vida social se ha visto afectada por tener que cuidar de su familiar?	◯ Nunca<br>◯ Casi nunca<br>◯ A veces<br>◯ Frecuentemente<br>◯ Casi siempre
S52513 ¿Se siente incómodo para invitar amigos a casa, a causa de su familiar?	◯ Nunca<br>◯ Casi nunca<br>◯ A veces<br>◯ Frecuentemente<br>◯ Casi siempre
S52514 ¿Cree que su familiar espera que usted le cuide, como si fuera la única persona con la que puede contar?	◯ Nunca<br>◯ Casi nunca<br>◯ A veces<br>◯ Frecuentemente<br>◯ Casi siempre

S52515 ¿Cree que no dispone de dinero suficiente para cuidar a su familiar además de sus otros gastos?	◯ Nunca<br>◯ Casi nunca<br>◯ A veces<br>◯ Frecuentemente<br>◯ Casi siempre
S52516 ¿Siente que será incapaz de cuidar a su familiar por mucho más tiempo?	◯ Nunca<br>◯ Casi nunca<br>◯ A veces<br>◯ Frecuentemente<br>◯ Casi siempre
S52517 ¿Siente que ha perdido el control sobre su vida desde que la enfermedad de su familiar se manifestó?	◯ Nunca<br>◯ Casi nunca<br>◯ A veces<br>◯ Frecuentemente<br>◯ Casi siempre
S52518 ¿Desearía poder encargar el cuidado de su familiar a otras personas?	◯ Nunca<br>◯ Casi nunca<br>◯ A veces<br>◯ Frecuentemente<br>◯ Casi siempre
S52519 ¿Se siente inseguro acerca de lo que debe hacer con su familiar?	◯ Nunca<br>◯ Casi nunca<br>◯ A veces<br>◯ Frecuentemente<br>◯ Casi siempre
S52520 ¿Siente que debería hacer más de lo que hace por su familiar?	◯ Nunca<br>◯ Casi nunca<br>◯ A veces<br>◯ Frecuentemente<br>◯ Casi siempre
S52521 ¿Cree que podría cuidar de su familiar mejor de lo que lo hace?	◯ Nunca<br>◯ Casi nunca<br>◯ A veces<br>◯ Frecuentemente<br>◯ Casi siempre
S52522 En general: ¿Se siente muy sobrecargado por tener que cuidar de su familiar?	◯ Nunca<br>◯ Casi nunca<br>◯ A veces<br>◯ Frecuentemente<br>◯ Casi siempre
Suma Zarit REDCap	__________________________
Este resultado clasifica al cuidador en:	◯ Ausencia de sobrecarga (≤46)<br>◯ Sobrecarga ligera (47-55)<br>◯ Sobrecarga intensa (≥56)

**S635 EQ5D5L CUIDADOR. © EuroQol Research Foundation. EQ-5D™ is a trade mark of the EuroQol Research Foundation.**	
S6351 Haga clic en UNA casilla, la que mejor describe su MOVILIDAD en el día de HOY.	◯ No tengo problemas para caminar-1<br>◯ Tengo problemas leves para caminar-2<br>◯ Tengo problemas moderados para caminar-3<br>◯ Tengo problemas graves para caminar-4<br>◯ No puedo caminar-5
S63511 Resultado Movilidad	______________________________
S6352 Haga clic en UNA casilla, la que mejor describe su AUTO-CUIDADO en el día de HOY.	◯ No tengo problemas para lavarme o vestirme-1<br>◯ Tengo problemas leves para lavarme o vestirme-2<br>◯ Tengo problemas moderados para lavarme o vestirme-3<br>◯ Tengo problemas graves para lavarme o vestirme-4<br>◯ No puedo lavarme o vestirme-5
S63521 Resultado Auto-Cuidado	______________________________
S6353 Haga clic en UNA casilla, la que mejor describe su capacidad para ACTIVIDADES COTIDIANAS en el día de HOY.	◯ No tengo problemas para realizar mis actividades cotidianas-1<br>◯ Tengo problemas leves para realizar mis actividades cotidianas-2<br>◯ Tengo problemas moderados para realizar mis actividades cotidianas-3<br>◯ Tengo problemas graves para realizar mis actividades cotidianas-4<br>◯ No puedo realizar mis actividades cotidianas-5
S63531 Resultado Actividades Cotidianas	______________________________
S6354 Haga clic en UNA casilla, la que mejor describe su DOLOR/MALESTAR en el día de HOY.	◯ No tengo dolor ni malestar-1<br>◯ Tengo dolor o malestar leve-2<br>◯ Tengo dolor o malestar moderado-3<br>◯ Tengo dolor o malestar fuerte-4<br>◯ Tengo dolor o malestar extremo-5
S63541 Resultado Dolor/Malestar	______________________________
S6355 Haga clic en UNA casilla, la que mejor describe su ANSIEDAD/DEPRESIÓN en el día de HOY.	◯ No estoy ansioso ni deprimido-1<br>◯ Estoy levemente ansioso o deprimido-2<br>◯ Estoy moderadamente ansioso o deprimido-3<br>◯ Estoy muy ansioso o deprimido-4<br>◯ Estoy extremadamente ansioso o deprimido-5
S63551 Resultado Ansiedad/Depresión	______________________________
S6366 Estado de salud (España)	______________________________<br>(Anote la secuencia de números resultante de las cinco (5) respuestas: S73511 /S73521 /S73531 /S73541 /S73551.)

S6367 Nos gustaría conocer lo buena o mala que es su salud HOY.
La escala está numerada del 0 al 100.
100 representa la MEJOR salud que usted se pueda imaginar.
0 representa la PEOR salud que usted se pueda imaginar.
Haga clic en la escala para indicar cuál es su estado de salud HOY.

0 50 100

(Place a mark on the scale above)
